# A pH‐Switchable System for On‐Demand Solar Hydrogen Production

**DOI:** 10.1002/cssc.202500029

**Published:** 2025-04-06

**Authors:** Alberto Bianco, Francesca Mancini, Giacomo Bergamini

**Affiliations:** ^1^ Department of Chemistry “Giacomo Ciamician” University of Bologna Via Piero Gobetti 85 40129 Bologna Italy

**Keywords:** hydrogen evolution, on‐demand, ruthenium oxide, solar energy storages

## Abstract

Artificial solar‐to‐fuel conversion is a pivotal pathway toward a sustainable energy future. Molecular hydrogen H_2_, with its clean energy potential, emerges as a promising candidate to replace fossil fuels. Nevertheless, the intermittent nature of solar irradiation presents a formidable obstacle. Inspired by natural photosynthesis, a well‐known three‐component system is employed to decouple light absorption and hydrogen evolution. The system utilizes [Ru(bpy)_3_]^2+^, triethanolamine, and methyl viologen to store solar energy as reduced viologen (MV^•+^). By controlling pH, this stored energy can be efficiently released to produce hydrogen on demand. The system demonstrates superior efficiency compared to platinum‐based catalysts, along with remarkable reversibility, cyclability, and stability. This work significantly advances solar‐to‐hydrogen conversion, providing a promising solution for the intermittent nature of solar energy and paving the way to a sustainable energy future.

## Graphical abstract

The proposed window‐ledge system harnesses solar energy by converting it into stored electrons. These can be then efficiently used on‐demand to produce H_2_ through pH‐controlled reactions, surpassing the performance of platinum‐based catalysts. The approach offers a highly reversible, cyclable, and stable solution for solar‐to‐hydrogen conversion, addressing the intermittent nature of solar energy and contributing to a sustainable energy future.

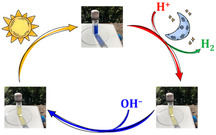

## 1 Introduction

The photochemical conversion of solar energy into fuels and chemicals is a cornerstone of future energy strategies, offering the most promising pathway for decarbonization.^[^ ^1^, ^2^ ^]^

Specifically, molecular hydrogen (H_2_) is considered the ideal solar fuel due to its role as a clean energy vector. Unlike fossils, its complete combustion produces only water, eliminating CO_2_ emissions and promoting a closed‐loop energy cycle.^[^ ^3^, ^4^, ^5^, ^6^ ^]^ However, solar‐driven photochemistry faces a significant challenge: the inherent mismatch between diurnal light availability and continuous energy demand.^[^ ^7^ ^]^ This limits its suitability for large‐scale and industrial processes.^[^ ^8^, ^9^ ^]^ Inspired by natural photosynthesis, which generates reactive photoredox equivalents that can be stored and regenerated as needed,^[^ ^10^ ^]^ a method to overcome this limitation consists of decoupling the light absorption and hydrogen evolution steps through the introduction of an electron storage intermediate. In this way, solar energy harvesting and hydrogen production phases can be separated over time. This design allows for the “on‐demand” generation of H_2_ in the absence of light, triggered by an external stimulus.

While coupling photovoltaic cells, batteries, and water electrolyzers can theoretically create such systems by simply inserting a circuit breaker,^[^ ^11^ ^]^ energy losses at each stage inherently limit overall energy conversion efficiency and hydrogen evolution rates.^[^ ^12^, ^13^ ^]^ As a viable alternative to multistep photovoltaic H_2_ generation, several multicomponent heterogeneous systems have been developed. These systems store electrons in reduced molecules or semiconductors and employ a specific stimulus to prompt H_2_ evolution in the dark. Common stimuli reported in the literature^[^ ^14^ ^]^ include the addition of a hydrogen evolution catalyst (HEC), typically Pt,^[^ ^15^ ^]^ the addition of a hydrogen atom source,^[^ ^16^, ^17^, ^18^, ^19^, ^20^, ^21^ ^]^ or the H^+^ concentration.^[^ ^22^, ^23^, ^24^ ^]^ However, these approaches frequently exhibit poor controllability and reversibility, hindering their cyclability. Additionally, they bring to large by‐product accumulation, resulting in a low atom economy. All these aspects prevent the sustainability of such systems, so their practical application. In aqueous media, the latter approach listed above corresponds to pH regulation, the most reversible and “clean” process, producing no wastes other than the already present water and counterions.

**Scheme 1:**
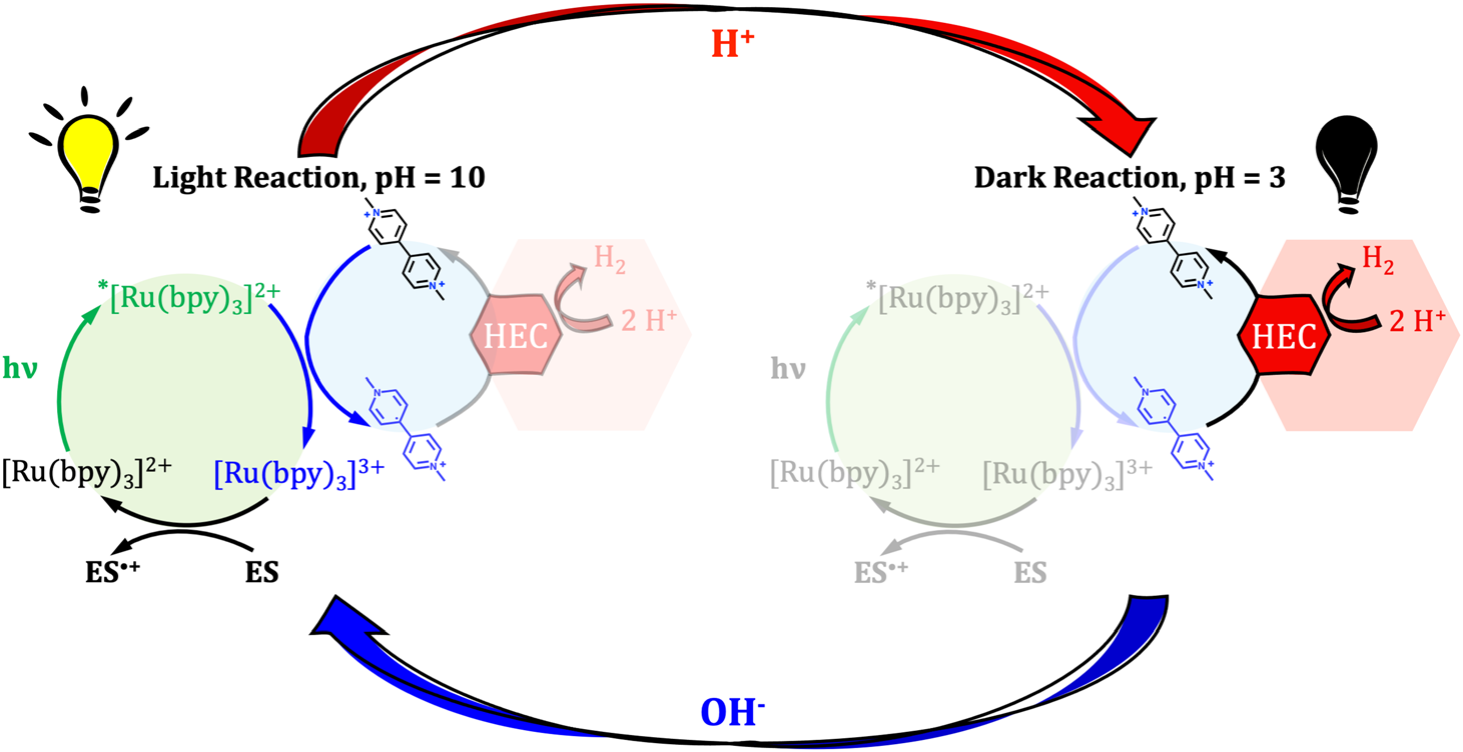
Representation of the decoupled TCS employed in this study. Left side represents the photocycle which, in alkaline media, brings to the MV^•+^ accumulation; right side represents the H_2_ evolution phase, which takes place in acidic media.

Herein, we propose to finely control the archetypal three‐component system (TCS) in which the photosensitizer ruthenium tris‐bipyridyl ([Ru(bpy)_3_]^2+^), upon excitation, transfers one electron to methyl viologen (MV^2+^) generating [Ru(bpy)_3_]^3+^ and MV^•+^.^[^ ^25^ ^]^ An electron source (ES) then restores the starting [Ru(bpy)_3_]^2+^, closing the photocatalytic cycle and enabling the accumulation of reduced methyl viologen. By carefully selecting the electron source, we can control whether the photocycle operates in an alkaline or acidic environment. Specifically, employing aliphatic amines as electron sources^[^ ^26^ ^]^ confines the photosystem efficient cycling to alkaline pH ranges (**Scheme** **1**, left side).

The reduced methyl viologen generated with this method is thus indefinitely stable in an anaerobic alkaline environment,^[^ ^27^, ^28^ ^]^ making it one of the best chemical storages of “ready‐to‐use” electrons. Then, in acidic media, MV^•+^ is able to prompt the proton reduction in the presence of a proper HEC (Scheme 1, right side), which we demonstrated to be ruthenium (IV) oxide.^[^ ^29^ ^]^ Relying on that, we developed a fully reversible two‐stage system in which the “light reaction” is the formation and accumulation of MV^•+^ in an alkaline medium, and the “dark reaction” is the discharge of these stored electrons on RuO_2_ nanostructure after acidification, resulting in H_2_ production. The system, as composed, combines high stability under irradiation with promising hydrogen evolution yields, while the modular design based on molecular subunits allows independent chemical tuning and optimization. This design paves the way for future automated and scalable implementations.

## 2 Results and Discussion

As we previously demonstrated, the application of commercial RuO_2_ nanostructures as HEC in a classic TCS, based on the [Ru(bpy)_3_]^2+^/MV^2+^ photoinduced electron transfer and an ES, is able to generate H_2_ in a continuous regime with a rate of 0.137 mol h^−1^ g^−1^ and a 6.8% apparent quantum efficiency.^[^ ^29^ ^]^ More specifically, to achieve the correct balance between the efficiencies of the TCS and the hydrogen evolution reaction (HER), the complete system needs an electron source which is easily oxidized at slightly acidic pH values, such as EDTA·2Na or L‐Cysteine.

While most electron sources employed in photocatalytic cycles function optimally under alkaline conditions, catalysts for hydrogen evolution exhibit higher activities in acidic environments. This discrepancy in pH ranges for the electron source and the catalyst hinders the efficient and continuous production of hydrogen.

By decoupling the photochemical process from catalytic H_2_ evolution, we eliminate the necessity for specific electron sources. This broadened scope allows us to consider molecules that are oxidized only upon complete deprotonation, such as aliphatic amines, which operate optimally in a basic environment.

Considering these factors, we selected triethanolamine (TEOA) as the electron donor.^[^ ^25^ ^]^ This choice was motivated by its stability, affordability, and ability to generate reducing products via primary oxidation processes, as demonstrated in early experiments on this system.^[^ ^30^ ^]^

To quantify the initial photoinduced electron transfer from the excited ruthenium complex to MV^2+^, we measured MV^•+^ formation upon irradiating a 2 mL aqueous TCS solution (containing [Ru(bpy)_3_]^2+^ at concentrations ranging from 6.8 to 67.6 μm, 5.0 mm MV^2+^, 0.1 m TEOA, 0.1 mg of RuO_2_, at pH = 10) with a 460 nm LED. While maintaining constant concentrations of all other components, we systematically increased the [Ru(bpy)_3_]^2+^ concentration to adjust the fraction of absorbed light at 460 nm from 20 to 90%. The MV^•+^ concentration was precisely quantified throughout the irradiation process by measuring the solution's absorbance at 732 nm and applying the Beer–Lambert–Bouguer law (MV^•+^ *ε*_732nm_ = 2900 m ^−1^ cm^−1^ in water, see Figure S3, Supporting Information). To prevent reoxidation of the viologen radical cation by molecular oxygen, the solution was maintained under strictly anaerobic conditions. A custom‐made “freeze‐pump‐thaw” cuvette was used for these irradiation experiments (see Supporting Information for detailed procedure).

**Figure 1:**
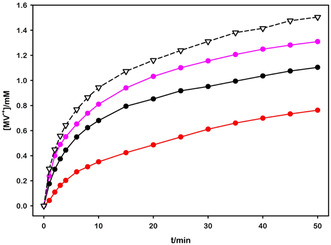
MV^•+^ concentration obtained upon 460 nm irradiation of deaerated 2 mL aqueous TCS solution (5.0 mm MV^2+^, 0.1 m TEOA, 0.1 mg of RuO_2_, at pH = 10; 1.00 cm pathlength). Red line refers to 6.8 μm, black line to 27.0 μm, and pink line to 67.6 μm Ru complex concentration (20, 60, and 90% absorbed light at 460 nm, respectively). Dashed black line with triangles corresponds to the MV^•+^ photoformation obtained irradiating the 27 μm solution in a 0.20 cm pathlength cell.

The observed variations (**Figure** **1**) clearly demonstrate a correlation between the photons absorbed and the formation and accumulation of MV^•+^. However, the relationship was not linear with concentration, and we found that at an absorbance of 0.4 at 460 nm (27.0 μm concentration of Ru complex, 60% light absorption), the maximum rate of photoproduction was nearly achieved. Given the cost of the ruthenium complex, we opted to operate at this concentration.

Furthermore, the experiments depicted in Figure 1 demonstrate that, regardless of Ru concentration (and, consequently, absorbed light fraction), MV^•+^ formation slows down at a relatively low level compared to the total MV^2+^ present. This result indicates that only a fraction of the viologen is reduced during irradiation. This behavior is attributed to the progressive consumption of the quencher MV^2+^, but mostly to the inner filter effect, wherein reduced viologen absorbs light at the irradiation wavelength, hindering further photoreduction (see Figure S4, Supporting Information). While this phenomenon may initially appear detrimental due to partial electron storage, it actually enhances system stability. At millimolar MV^•+^ concentrations, its strong absorption across the visible spectrum provides an extremely efficient self‐protection mechanism against photodegradation. Obviously, this effect is path length dependent. To investigate the impact of path length, we repeated the experiment with [Ru(bpy)_3_]^2+^ 27.0 μm concentration using a 0.20 cm cuvette. This allowed us to reduce the inner filter effect caused by reduced viologen, enabling the storage of a larger quantity of MV^•+^. The black dashed curve shown in Figure 1 clearly demonstrates that this strategy was effective. However, this leads to a lower self‐protection effect and a smaller amount of reduced viologen per unit of irradiated surface. Nonetheless, our findings indicate that larger surface areas can be irradiated in thinner layers, facilitating higher storage of electrons.

This design flexibility enables system optimization for various applications. Specifically, for high‐irradiance applications, the protection mechanism should be maximized to ensure on‐demand system stability. Conversely, in low‐irradiance environments, this self‐protecting mechanism appears to be unnecessary, therefore prioritizing storage maximization.

**Figure 2:**
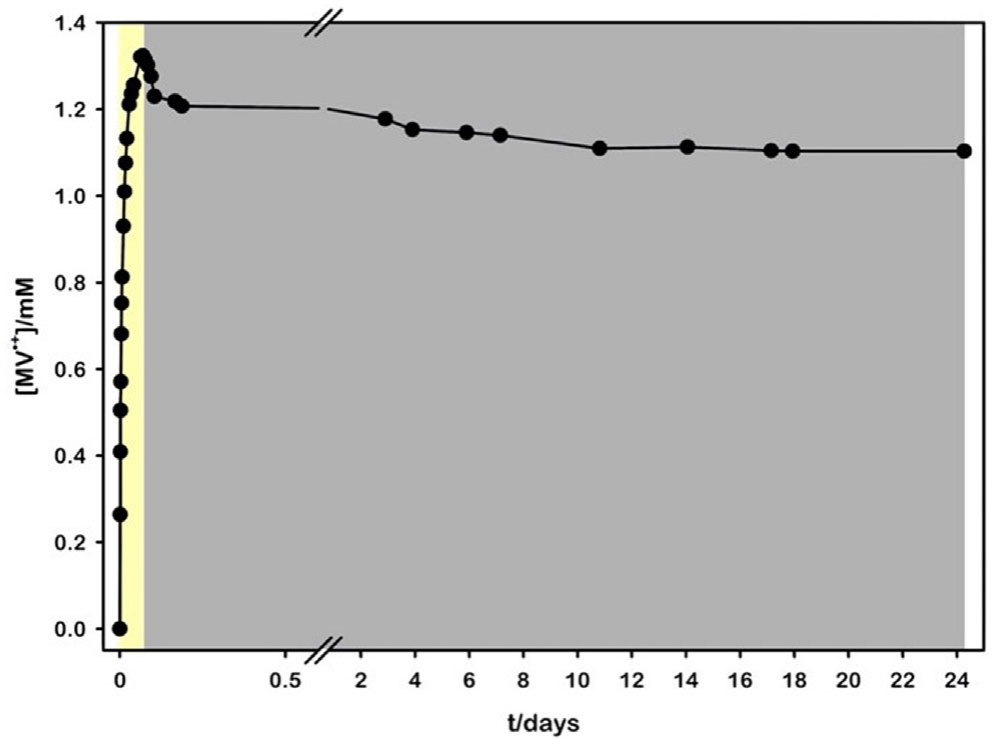
MV^•+^ concentration obtained upon 460 nm irradiation of deaerated TCS solution (yellow box, 90 min irradiation) and subsequent storage in dark (grey box).

To demonstrate the effective decoupling and practical application of the proposed system, we assessed the long‐term stability of the viologen radical cation in the same conditions described above. **Figure** **2** presents the time‐dependent MV^•+^ concentration in the irradiated solution, demonstrating a less than 10% decrease over 24 days. This decrease is most likely attributable to a minor leak in the cuvette stopcock, allowing atmospheric oxygen to slowly diffuse into the system. This effect is observed even in the absence of an HEC.

The experiment described above confirms the rapid production of MV^•+^ under these conditions and proves its remarkable stability, identifying this species as perfect electron storage in aqueous solution.

**Figure 3:**
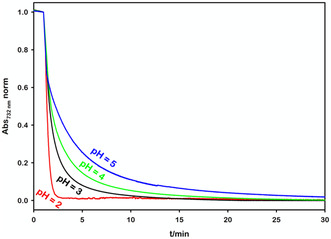
Kinetic evolution of normalized 732 nm absorbance (attributable to MV^•+^) in the presence of RuO_2_ at different final pH values; HCl injected at *t* = 1 min.

The next step is to determine the optimal experimental conditions for transferring stored electrons from the reduced viologen to the catalyst, thereby promoting H_2_ synthesis. To that end, we placed 2.0 mL of a solution composed of 27 μm [Ru(bpy)_3_]^2+^, 5.0 mm MV^2+^, 0.1 m TEOA, and 0.1 mg of RuO_2_, at pH = 10 in a quartz cuvette (optical pathlength 1.00 cm) sealed with a rubber septum. After degassing the solution with vigorous argon bubbling (Ar flow 50 mL min^−1^, total time 15 min), we irradiated it for 50 min with the previously described setup. Once obtained the deep‐blue coloration, we followed the absorbance at 732 nm before and after the addition of different amounts of hydrochloric acid aqueous solution (HCl, 6 m). To investigate the pH‐dependent discharge behavior, four replicate experiments were conducted. The amount of added HCl was systematically increased (from 30 to 40 μL) using a gas‐tight syringe through the rubber septum and monitoring the decrease of reduced viologen at pH values of 5, 4, 3, and 2. The results of these experiments are presented in **Figure** **3**.

The acidification results in different rates of disappearance of the characteristic deep‐blue color along with the complete recovery of the initial absorption spectrum, confirming that a decrease in pH correlates with a significant increase in the electron discharge rate on RuO_2_. This effect is particularly pronounced when the pH drops from 3 to 2. To elucidate the underlying mechanism, we replicated these measurements in the absence of the HEC. We found that at pH ≥ 3, the catalyst is exclusively responsible for the viologen reoxidation. However, at pH 2, MV^•+^ depletion occurs even without the catalyst (Figure S5, Supporting Information). This behavior is likely attributable to the protonation of reduced viologen, leading to its degradation (see Supplementary Information for the detailed mechanism).^[^ ^27^ ^]^

Following these results, we keep adding 35 μL of 6 m HCl, obtaining a final pH = 3 in all the following experiments.

However, decolorization alone is not sufficient to prove the actual H_2_ production, and therefore, direct measurement of the quantity of molecular hydrogen produced was carried out using gas chromatography. The measurements unequivocally demonstrate H_2_ evolution.

Given the decoupled nature of our proposed system, traditional metrics like solar‐to‐hydrogen efficiency and apparent quantum yield are less relevant. In particular, the efficiency of photoreduced species production in the light reaction is strongly influenced by the initial bimolecular quenching process. To disengage the two aspects, we defined an efficiency solely for the “dark reaction” ($\eta_{\text{DR}}$). Since MV^•+^ represents a stored high‐energy electron, its conversion to hydrogen at the catalyst interface is essentially an electron‐to‐molecule process, regardless of the quantity or generation method of reduced species. Therefore, we can draw a parallel with the Faradaic efficiency commonly employed in electrochemical systems^[^ ^31^ ^]^

(1) $$\eta_{\text{DR}}=\frac{2\cdot n{\text{ mol}}_{H_{2}}}{n{\text{ mol}}_{{\text{MV}}^{•+}}}\times 100$$

Using this approach (quantification procedure reported in the Supporting Information), we could estimate a 35% efficiency from photogenerated MV^•+^ to evolved H_2_.

**Figure 4:**
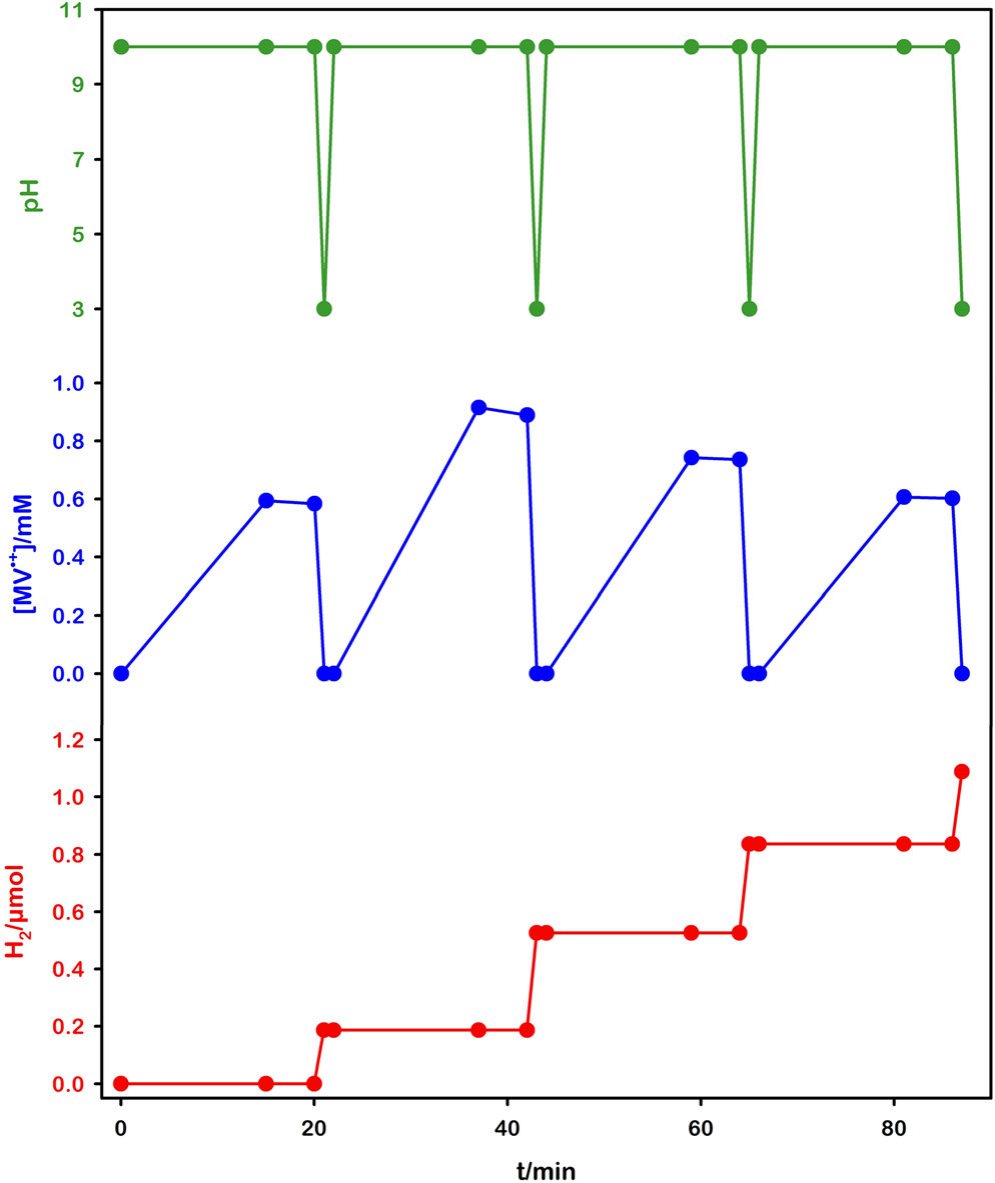
pH variations (green), MV^•+^ concentration (blue), and evolved H_2_ (red) over our on‐demand system cyclic operation.

Furthermore, to test the system recyclability, after H_2_ production at acidic pH, a return to basic pH was achieved by adding NaOH. The addition of base alone does not lead to any variation in the absorption spectrum of the solution, and thus, of the component integrity. Upon reirradiation of the solution, the formation of reduced viologen is observed again. Direct irradiation of the acidic solution does not induce a color change. Although the excited ruthenium complex photoreduces the viologen, this electron is immediately transferred back to the oxidized photosensitizer, restoring the pristine Ru complex and not leading to MV^•+^ accumulation. This is due to the complete protonation of TEOA at such pH values, which prevents it from acting as an electron source to close the photocatalytic process. This acid‐base cycle can be repeated several times, obtaining subsequent on‐demand H_2_ production from the deep‐blue reduced solution. **Figure** **4** shows the pH variations, the MV^•+^ production (measured by the absorption spectra) and the moles of H_2_ produced (measured by headspace GC‐MS) over four irradiation‐storage‐H_2_ production cycles.

The different amounts of reduced viologen obtained in the various cycles depicted above are solely due to slightly different experimental conditions, and not to degradation of components or parasitic reactions. Specifically, we achieved cycles with identical number of reduced species, but we report this experiment to highlight the direct correlation between the amount of reduced viologen and H_2_ formed.

The system demonstrated remarkable reversibility and stability. Hydrogen evolution is directly correlated with MV^•+^ photoproduction, and notably, the catalyst maintains its 35% efficiency throughout multiple cycles (see Table S1, Supporting Information for comprehensive results). To ensure that this value is not influenced by the nature of the electron source, we tested the same system using l‐cysteine as an electron donor. The results demonstrated that using the same pH switch (10–3), the dark reaction efficiency remained highly reproducible at 35%, regardless of the rate of the light reaction.

Additionally, we tested the same system replacing RuO_2_ with platinum (see Figure S7, Supporting Information), which is still considered the benchmark as HEC.^[^ ^32^ ^]^ This resulted in a significant reduction in efficiency for the conversion of MV^•+^ to H_2_, down to only 12%. As reported in our previous paper, PtNps not only demonstrated a lower efficiency and a limited versatility compared to RuO_2_,^[^ ^29^ ^]^ but also are detrimental to the stability of the other molecular components since Pt promotes the hydrogenation of aromatic moieties.

The stability of the ruthenium complex was assessed using absorption spectroscopy (see Figure S8, Supporting Information), while MV^2+^ concentration was indirectly quantified by measuring the quenching of the ruthenium complex luminescence lifetime. Under our experimental conditions, MV^2+^ was the sole quencher of the excited ruthenium complex. Consequently, monitoring the ruthenium luminescence lifetime provides a sensitive method for determining the pristine viologen concentration. Continuous operation at pH 7 revealed that methyl viologen was consumed exclusively in the presence of platinum nanoparticles (see Figure S9 and Table S3, Supporting Information), suggesting its hydrogenation at the platinum surface.^[^ ^33^ ^]^

Moreover, we tested our system using a solar simulator. Results indicate that, as expected, MV^•+^ production occurred at a slower rate due to the lower irradiation intensity compared to the 460 nm LED. However, once the reduced viologen was formed, the efficiency for H_2_ production remained constant at around 33% (see Table S2, Supporting Information). To assess real‐world performance, we also conducted irradiations on the laboratory windowsill on a sunny day. As shown in Figure S10, Supporting Information, solar irradiation led to the characteristic blue coloration of reduced MV^•+^, and the addition of acid resulted in decolorization and H_2_ formation. Again, without precise control over the photons absorbed by the system, it is difficult to provide an absolute production rate.

However, we can conclude that, regardless of the light source used for MV^2+^ photoreduction, the efficiency of the light reaction varies, while the $\eta_{\text{DR}}$ remains constant. This allows for flexible modulation of the system based on specific requirements such as accumulation time, volume, and photoreactor configuration. Additionally, the key point of this study is the electron‐storage capability of MV^•+^ and its subsequent on‐demand conversion to H_2_. This radical cation can be produced through multiple pathways, including nonphotochemical methods, allowing for the independent manipulation of electron storage and hydrogen evolution processes.

## 3 Conclusion

In summary, we have demonstrated a pH‐switchable system for on‐demand solar hydrogen production that efficiently decouples the photochemical light absorption phase from the catalytic hydrogen evolution step. By leveraging the well‐known [Ru(bpy)_3_]^2+^/MV^2+^/TEOA TCS, we have developed a two‐stage process where the formation and accumulation of reduced viologen (MV^•+^) is promoted by visible irradiation under alkaline conditions (light reaction), while hydrogen production is triggered by subsequent acidification of the solution (dark reaction). Using affordable reagents, such as hydrochloric acid and sodium hydroxide, as pH stimulus to control hydrogen evolution offers a neat, reversible, and environmentally sustainable method, leading to no by‐product production besides sodium chloride.

The reported results highlight the high efficiency of reduced viologen storage, coupled with its exceptional stability in alkaline conditions. This advantage of decoupling storage and hydrogen production in acidic environments, along with the self‐protection effect of MV^•+^, makes this system applicable even under high irradiance conditions.

The system described in this work represents a highly advanced starting point to address the challenge of intermittent energy that can be implemented both by engineering its configuration and control and/or by integrating it with electrochemical and photoelectrochemical systems.

## Supporting Information

Materials and Methods sections, reduced viologen photoaccumulation and Long‐term Storage experimental details, reduced methyl viologen molar absorption spectrum, pH‐dependant MV^•+^ stability tests; MV^•+^ degradation pathway, H2 production measurements and quantification with different HECs, MV2+ stability during H2 evolution, and window‐ledge irradiation pictures can be found in the Supporting Information. The authors have cited additional references within the Supporting Information.^[34,35]^

## Conflict of Interest

The authors declare no conflict of interest.

## Supporting information

Supplementary Material
