# Supplementary material for: A pH‐Switchable System for On‐Demand Solar Hydrogen Production
Source: ChemSusChem. 2025 Apr 6;18(12):e202500029. doi: 10.1002/cssc.202500029 (PMC12175054; doi:10.1002/cssc.202500029)
Supplement: Supplementary file 1 — Supplementary Material [file CSSC-18-e202500029-s001.pdf]

Supporting information for

A pH-Switchable System for On-Demand Solar Hydrogen Production

Alberto Bianco, Francesca Mancini, and Giacomo Bergamini

Department of Chemistry ‘‘Giacomo Ciamician’’

University of Bologna

Via Piero Gobetti 85, 40129 Bologna, Italy

Via Francesco Selmi 2, 40126 Bologna, Italy

Corresponding author’s e-mail address: giacomo.bergamini@unibo.it

Materials

High-purity triethanolamine (TEOA, ≥ 99.0%), fuming hydrochloric acid (37% wt), sodium hydroxide (≥ 99.9%), Chloroplatinic acid (H_2PtCl_6, 99.995% trace metal basis) and Poly(vinyl alcohol) (PVA, MW ≈ 130 000, > 99% hydrolyzed) were purchased from Merck and used with no further purification. Tris(2,2′-bipyridyl)ruthenium(II) chloride hexahydrate ($[Ru(bpy)_3]Cl_2{\cdot}6H_2O$, 99.95%) was purchased from Merck and re-crystallized tree times from methanol. 1,1′-Dimethyl-4,4′-bipyridinium dichloride ($MVCl_2$, >98%) was purchased from Merck and re-crystallized tree times from ethanol. Anhydrous ruthenium(IV) oxide (RuO_2, ≥99.9%) was purchased from STREM Chemicals and treated as previously reported.[1] Argon used for purging (filtered on Drierite™, 99.9995% purity) was supplied by SIAD. Type 1 ultrapure water was obtained with an Elga PURELAB® Classic UV apparatus. PVA-coated platinum nanoparticles were synthesized according to literature procedure.[2]

Methods

UV/Visible absorption spectra were recorded on a Perkin Elmer λ45 or Agilent Cary 300 double beam spectrophotometers, using custom-made quartz gas-tight cuvette with 1.00 cm or 0.20 cm path length. Emission lifetime decays were recorded on an Edinburgh Instruments FLS1000 spectrofluorometer equipped with a TCC2 electronic module for time-correlated single photon counting data acquisition (305 fs resolution), an EPL-450 pulsed diode laser and a cooled Hamamatsu R928 photomultiplier tube; using custom-made quartz gas-tight cuvette with 1.00 cm path length. All the decays were fitted with mono-exponential functions.

H_2 gas-chromatographic measurement were performed on an SRI 8610C gas-chromatograph equipped with a Thermal Conductivity Detector (TCD) and a Flame Ionization Detector (FID), both calibrated injecting 1 mL of standard gas mixtures of H_2 and CO_2 (5, 20, 100 and 1000 ppm of each component) supplied by *Air Liquide*. The separation was performed on a molecular sieve column under isothermal conditions (T_{Column} = 50°C) using argon as the carrier.

$MV^{\bullet+}$ Photoaccumulation and Long-term Storage

For testing the photoreduction of the viologen in presence of the HEC, we placed 2.0 mL of TCS aqueous solution ($[Ru(bpy)_3]^{2+}$ in different concentrations, MV^{2+} 5.0 mM and TEOA 0.1 M, 0.1 mg of RuO_2, pH = 10) in a custom made "freeze-pump-thaw" quartz cuvette (optical pathlength 1.00 cm, see Figure S1). The samples were then deaerated connecting this cuvette to a high-vacuum oil diffusion pump (maximum vacuum reached = $8.0 \cdot 10^{-7}$ mbar). To get rid of all oxygen dissolved in the solutions, the degassing procedure was repeated 5 time before closing the cuvette stopcock (equipped with three VITON® O-rings).

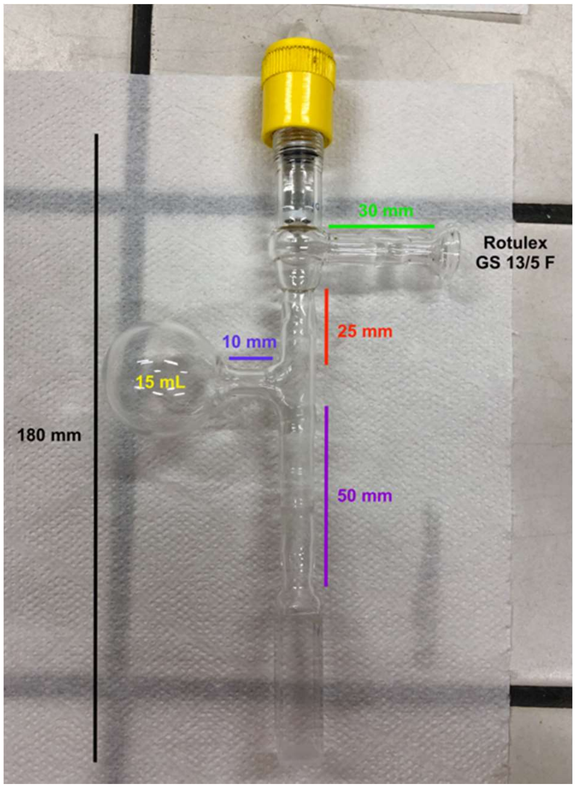

Figure S1. Custom made "freeze-pump-thaw" quartz cuvette with all the dimensions reported.

After degassing the samples, they were irradiated for 50 minutes under vigorous stirring at 460 nm using a high-power light-emitting diode (LED Engin LuxiGen™ LZ1-10B202-0000) operating at 600 mA and placed at a fixed distance of 5 cm from the cuvette quartz window.

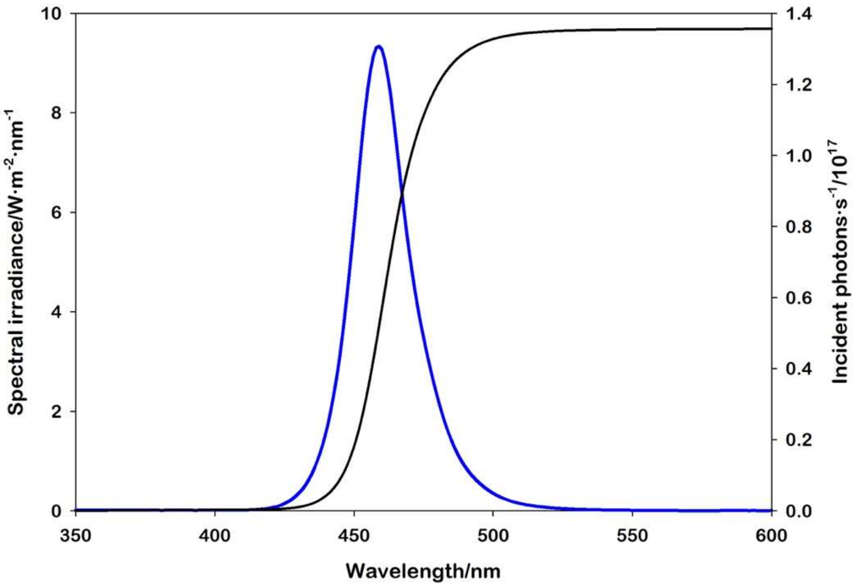

Figure S2. Spectral irradiance (blue line) of the 460 nm LED used in this study (LED Engin LuxiGen™ LZ1-10B202-0000, operating at 600 mA and 5 cm distance); total number of incident photons per second (dark grey line) at the photoreactor surface (S = 2.0 cm^2).

The $MV^{\bullet+}$ formation was then monitored by recording the absorption spectra of the solution at different irradiation times.

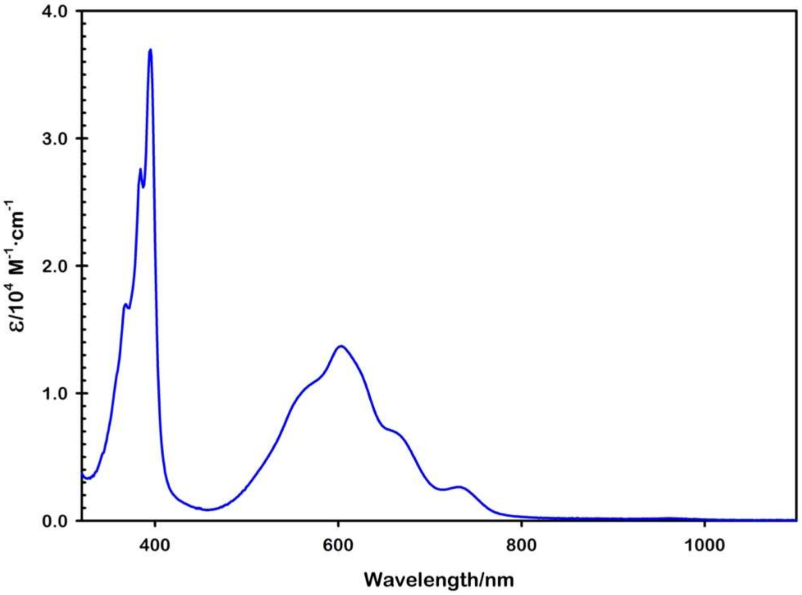

Figure S3. Molar absorption spectrum of $MV^{\bullet+}$ in water.

As stated in Main Text, the decrease of the $MV^{\bullet+}$ formation rate over time is attributable to the inner filter effect. To monitor this effect, we followed also the transmittance of the sample at 460 nm irradiation wavelength, as shown in Figure S3 (panel A).

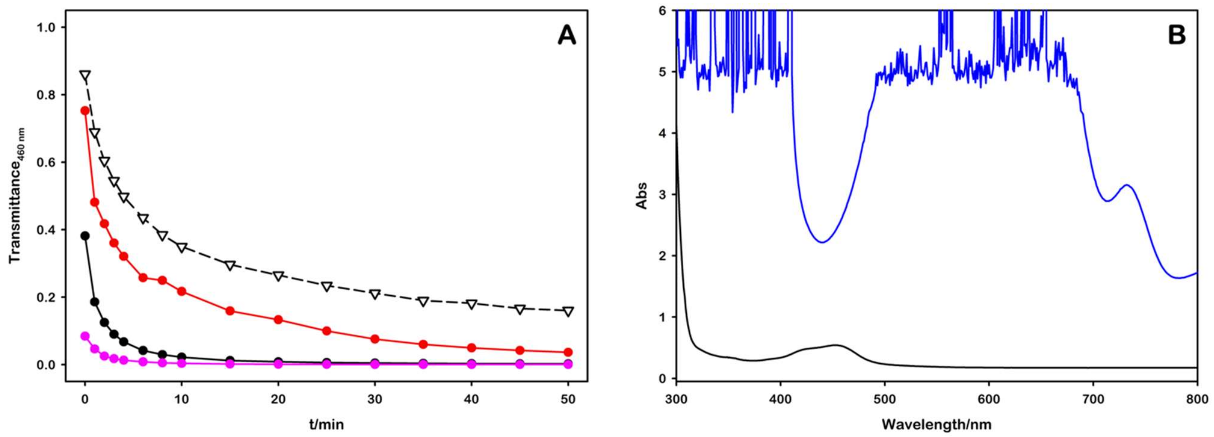

Figure S4. Panel A – 460 nm transmittance of deaerated TCS solution. Red line refers to 6.8 μM, black line to 27.0 μM and pink line to 67.6 μM Ru complex concentration. Dashed black line with triangles corresponds to the 460 nm transmittance of the 27μM solution in a 0.20 cm pathlength cell during irradiation. Panel B – Absorption spectra of the 27.0 μM Ru solution before (black line) and after 50 minutes irradiation at 460 nm (blue line), showing the inner filter effect.

pH-dependant $MV^{\bullet+}$ Stability

As reported in Main Text, to better investigate the pH-dependant discharge process, we repeated the acidic addition (pH = 2 and 3) to the $MV^{\bullet+}$ solution without RuO_2, as depicted in Figure S4.

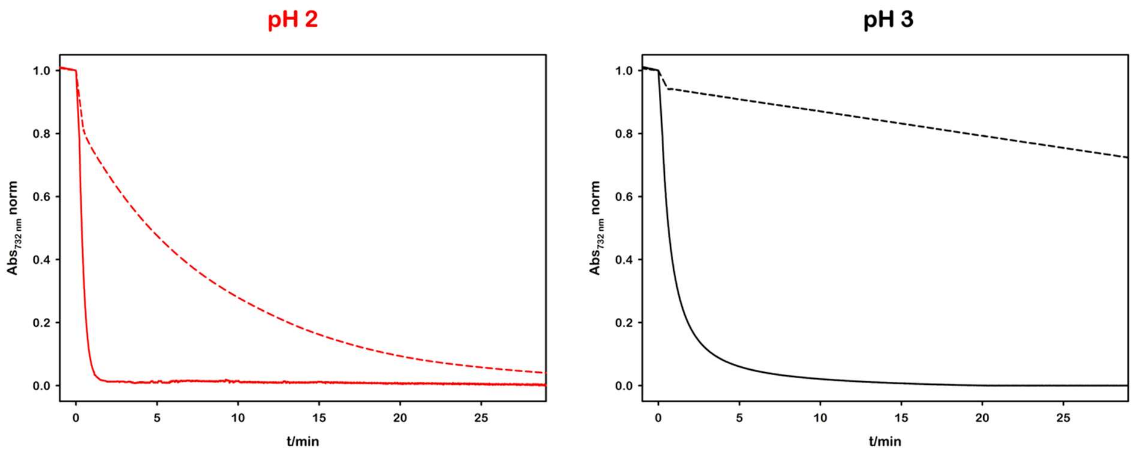

Figure S5. Kinetic evolution of 732 nm absorbance (attributable to $MV^{\bullet+}$) in presence (solid line) and in absence (dashed line) of RuO_2 at final pH values of 2 (left side, red lines) and 3 (right side, black lines); HCl injected at t = 1 min.

As it is evident, for pH = 2 the 732 nm absorbance drops rapidly also without RuO_2. This effect is due to the protonation of the viologen radical cation, which brings to its degradation according to the pathway reported in Figure S5.[3]

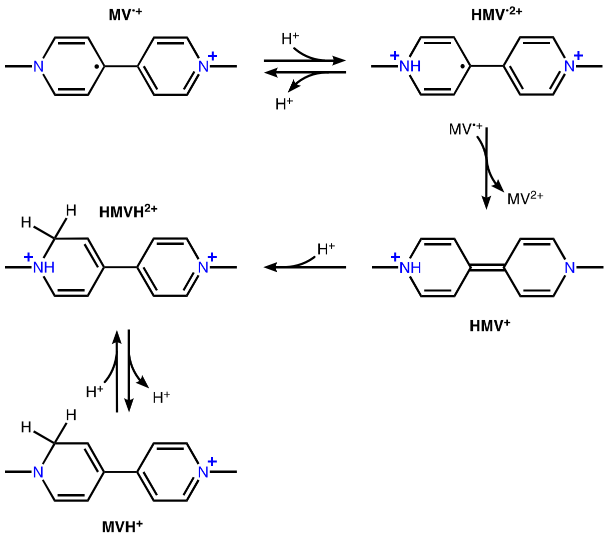

Figure S6. Irreversible degradation pathway of $MV^{\bullet+}$ induced by its protonation.

H_2 Production Measurements and Quantification

For exact quantification of evolved H_2, we placed 2.0 mL of TCS solution ($[Ru(bpy)_3]^{2+}$ 27.0 μM, MV^{2+} 5.0 mM and TEOA 0.1 M, 0.1 mg of RuO_2) in a quartz cuvette (optical pathlength 1.00 cm, total volume 3.5 mL) sealed with a rubber septum (Hamilton™ 3-Layer GC Septum, part # 76013). After degassing the solution with vigorous argon bubbling (Ar flow 50 mL·min^{-1}, total time 15 min), we irradiated the sample for 15 minutes. Then, we kept the solution in the dark for 5 minutes, thus checking the proper oxygen removal. After this step, we injected deaerated 6 M HCl aqueous solution through the septum with a gas-tight syringe, observing the rapid solution decolouration. Finally, we injected the cuvette headspace in the gas-chromatograph.

The GC measurement results with an area for the H_2 peak which is converted in H_2 headspace concentration (in ppm) using the aforesaid calibration.

$$A_{peak} \propto [H_2]_{ppm}$$

By knowing the whole headspace volume, it is possible to calculate the total volume of evolved H_2 after the acidification of the solution:

$$V_{H_2} = [H_2]_{ppm} \cdot V_{Headspace}\ [L]$$

Then, considering $T = 298.15\,K$ and $p = 1\,atm$, it is possible to convert the volume of evolved gas in moles using the following formula:

$$n\,mol_{H_2} = \frac{V_{H_2}}{24{,}45}\ [mol]$$

In which N_A is the *Avogadro constant* and $24{,}45$ are the litres occupied by a mole of perfect gas in the conditions described above.

After the steps reported above, the solution pH was restored to 10 by adding deaerated 6 M NaOH. Afterwards, the sample was purged with vigorous argon bubbling (Ar flow 50 mL·min^{-1}, total time 5 min) to clean the headspace from the H2 produced during the previous cycle, and re-irradiated, repeating the cycle.

Since the formation of an H_2 molecule from protons requires 2 distinct electrons, the dark reaction efficiency (η_{DR}) was calculated according to the following equation:

$$\eta_{DR} = \frac{2 \cdot n\,mol_{H_2}}{n\,mol_{MV^{\bullet+}}} \cdot 100$$

The same series of experiments have been repeated using PVA-coated platinum nanoparticles as HEC instead of RuO_2, obtaining the results depicted below:

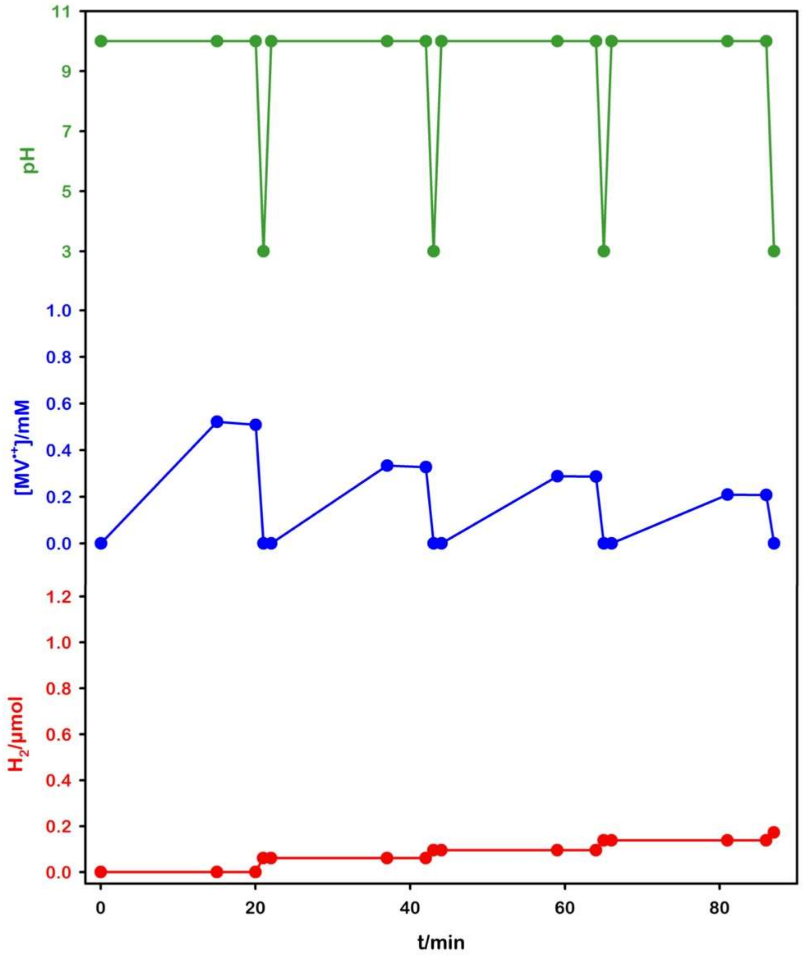

Figure S7. pH variations (green), $MV^{\bullet+}$ concentration (blue) and evolved H_2 (red) over our on-demand system cyclic operation using PtNps@PVA as the HEC.

The comprehensive results, along with conversion efficiency, are reported in the following table.

Table S1. $MV^{\bullet+}$ and H_2 μmol produced during each cycle (along with efficiency) of the proposed on-demand system using RuO_2 or PVA-coated PtNps as HECs (High-power LED irradiation). Cycle 0 refers to a headspace GC injection done before the first HCl addition, proving no H_2 evolution in alkaline conditions.

**Cycle number**	**RuO_2**			**PtNps@PVA**		
	μmol $MV^{\bullet+}$	μmol H_2	η_{DR}	μmol $MV^{\bullet+}$	μmol H_2	η_{DR}
**0**	1.086	0	NA	1.02	0	NA
**1**	1.086	0.189	34.7%	1.02	0.061	12.0%
**2**	1.887	0.345	36.6%	0.73	0.035	9.7%
**3**	1.754	0.317	36.2%	0.69	0.043	12.4%
**4**	1.592	0.246	30.9%	0.54	0.035	12.8%

The experiments with RuO_2 as HEC have been repeated following the same procedure reported above but irradiating the sample by directly exposing the cuvette to sunlight on the window ledge. This resulted in a lower $MV^{\bullet+}$ production, hence in a lower H_2 evolution, while maintaining the same efficiency of the whole process, as results summarized in Table S2 show.

Table S2. $MV^{\bullet+}$ and H_2 μmol produced during each cycle (along with efficiency) of the proposed on-demand system using RuO_2 as HEC (solar simulator). Cycle 0 refers to a headspace GC injection done before the first HCl addition, proving no H_2 evolution in alkaline conditions.

**Cycle number**	**RuO_2**		
	μmol $MV^{\bullet+}$	μmol H_2	η_{DR}
**0**	0.648	0	NA
**1**	0.648	0.104	32.1%
**2**	0.891	0.150	33.7%
**3**	0.425	0.071	33.4%

From these results, we calculated a *Solar-to-Hydrogen efficiency* η_{STH} equal to 0.08% for the 2nd cycle of this unoptimized system. This value was obtained considering 242 $kJ\cdot mol^{-1}$ as the H_2 molar enthalpy, 100 $mW\cdot cm^{-2}$ as the solar irradiance, 2.00 cm^2 as the irradiated surface, and 900 s as irradiation time.

$[Ru(bpy)_3]^{2+}$ Stability During H_2 Evolution

To evaluate the stability of the ruthenium complex during pH cycling, absorption spectra were acquired during the entire experiment, including after each GC injection. The spectrum acquired after the fourth pH cycle was corrected for the dilution factor of 1.14, which resulted from the addition of hydrochloric acid and sodium hydroxide solutions (initial volume: 2.00 mL, final volume: 2.28 mL). The resulting corrected spectra are shown in Figure S8.

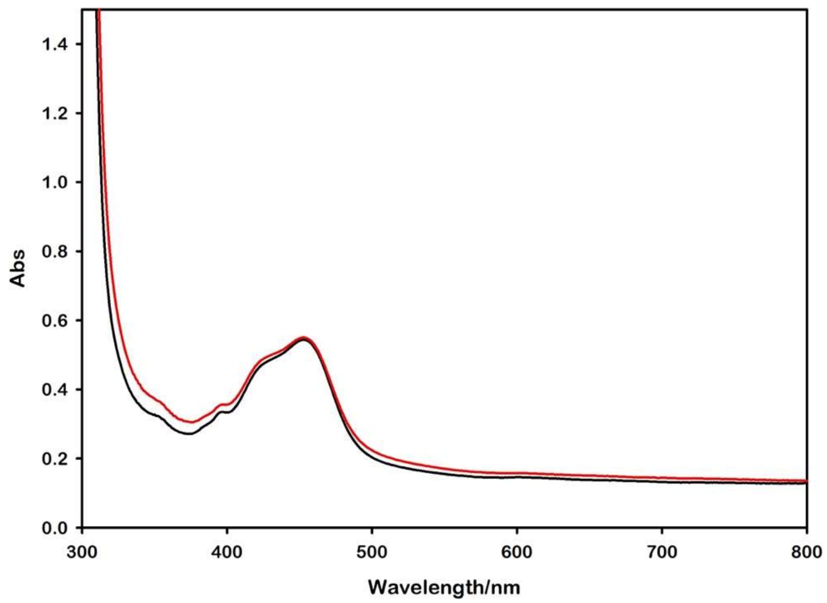

Figure S8. Absorption spectra of on-demand H_2 evolving system before irradiation (black line) and after the 4th GC injection (red line, corrected for dilution factor 1.14).

MV^{2+} Stability During H_2 Evolution

Since the hydrogen evolution reaction produces $H^{\bullet}$ radicals at the catalyst surface, these radicals can potentially hydrogenate organic substrates. While platinum, as a common HEC, promotes the hydrogenation of molecular components like the redox mediator methyl viologen, the use of RuO_2 effectively prevents this process. To demonstrate this, we measured the excited-state lifetime of ruthenium during system operation under various conditions: without any HEC, with RuO_2 and with PtNps@PVA.

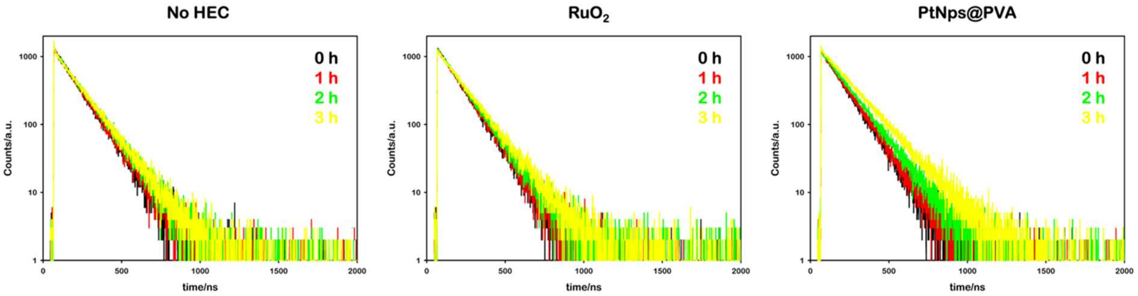

Figure S9. Lifetime decays of $[Ru(bpy)_3]^{2+}$ recorded at different time during on-demand H_2 evolving system operation without any HEC (left panel), with RuO_2 (central panel) and with PtNps@PVA (right panel).

As depicted in Figure S7, the lifetime of ruthenium increases during irradiation when using platinum. Based on the Stern-Volmer equation, which describes the quenching of an excited-state by dynamic processes, an increase in the excited-state lifetime indicates a decrease in the concentration of the quencher molecule.[4] Given that MV^{2+} is the sole quencher of the Ru complex excited-state under our conditions, the increase in the lifetime observed during operation using platinum HEC suggests that hydrogenation of the viologen dication is indeed occurring. In contrast, no significant change in lifetime is observed between RuO_2 and no HEC, indicating no degradation of the redox mediator MV^{2+} under these conditions, as reported in Table S3.

Table S3. Luminescence lifetimes of $[Ru(bpy)_3]^{2+}$ obtained by fitting the decays reported in Figure S7 with mono-exponential functions (χ^2 also reported).

Irradiation time / h	No HEC		RuO_2		PtNps@PVA	
	τ / ns	χ^2	τ / ns	χ^2	τ / ns	χ^2
**0**	123.4	1.0025	123.3	1.0005	123.5	1.0014
**1**	128.2	0.9976	125.7	0.9972	128.8	1.0005
**2**	137.8	1.0037	132.5	1.0004	144.6	1.0009
**3**	140.3	1.0012	141.1	1.0031	169.9	1.0015

Light and Dark reactions decoupling

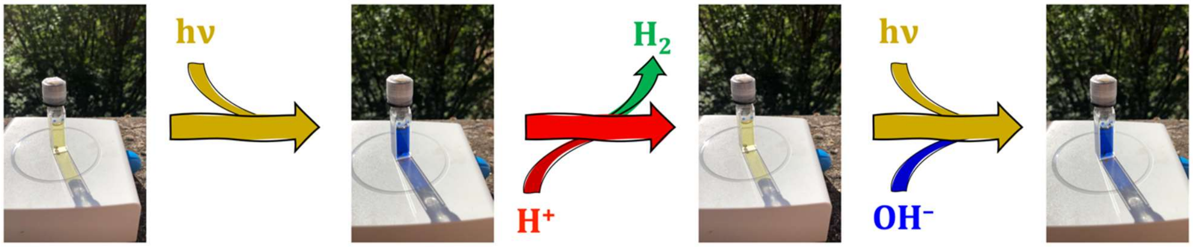

Figure S10. Schematic representation of light and dark reactions decoupling obtained by window-ledge irradiation.

References

[1] A. Bianco, A. Gradone, V. Morandi, G. Bergamini, *ACS Appl. Energy Mater.* **2023**, *6*, 6243–6250.

[2] N. Toshima, M. Kuriyama, Y. Yamada, H. Hirai, *Chem. Lett.* **1981**, *10*, 793–796.

[3] M. Venturi, Q. G. Mulazzani, M. Z. Hoffman, *Radiat. Phys. Chem.* **1984**, *23*, 229–236.

[4] P. Ceroni, Ed. , *The Exploration of Supramolecular Systems and Nanostructures by Photochemical Techniques*, Springer Netherlands, Dordrecht, **2012**.
